# A New Fluidized Bed Bioreactor Based on Diversion-Type Microcapsule Suspension for Bioartificial Liver Systems

**DOI:** 10.1371/journal.pone.0147376

**Published:** 2016-02-03

**Authors:** Juan Lu, Xiaoqian Zhang, Jianzhou Li, Liang Yu, Ermei Chen, Danhua Zhu, Yimin Zhang, LanJuan Li

**Affiliations:** State Key Laboratory for Diagnosis and Treatment of Infectious Diseases, Collaborative Innovation Center for Diagnosis and Treatment of Infectious Diseases, The First Affiliated Hospital, College of Medicine, Zhejiang University, Hangzhou, China; University of Cagliari, ITALY

## Abstract

A fluidized bed bioreactor containing encapsulated hepatocytes may be a valuable alternative to a hollow fiber bioreactor for achieving the improved mass transfer and scale-up potential necessary for clinical use. However, a conventional fluidized bed bioreactor (FBB) operating under high perfusion velocity is incapable of providing the desired performance due to the resulting damage to cell-containing microcapsules and large void volume. In this study, we developed a novel diversion-type microcapsule-suspension fluidized bed bioreactor (DMFBB). The void volume in the bioreactor and stability of alginate/chitosan microcapsules were investigated under different flow rates. Cell viability, synthesis and metabolism functions, and expression of metabolizing enzymes at transcriptional levels in an encapsulated hepatocyte line (C3A cells) were determined. The void volume was significantly less in the novel bioreactor than in the conventional FBB. In addition, the microcapsules were less damaged in the DMFBB during the fluidization process as reflected by the results for microcapsule retention rates, swelling, and breakage. Encapsulated C3A cells exhibited greater viability and CYP1A2 and CYP3A4 activity in the DMFBB than in the FBB, although the increases in albumin and urea synthesis were less prominent. The transcription levels of several CYP450-related genes and an albumin-related gene were dramatically greater in cells in the DMFBB than in those in the FBB. Taken together, our results suggest that the DMFBB is a promising alternative for the design of a bioartificial liver system based on a fluidized bed bioreactor with encapsulated hepatocytes for treating patients with acute hepatic failure or other severe liver diseases.

## Introduction

A bioartificial liver (BAL) support system that employs viable hepatocytes has been shown to provide temporary and important support, serving as a bridge to liver transplantation for patients with acute hepatic failure or other liver diseases [1]. A critical aspect of an effective BAL system is the configuration of the bioreactor, with the ideal bioreactor being able to maintain or even improve cell functions [2]. In addition, the bioreactor must provide sufficient bidirectional mass transport between cells and patients’ blood or plasma at a required scale for clinical use [3]. Currently, the most widely adopted bioreactor is based on a hollow fiber membrane, in which cells are cultured on capillary spaces, and the blood or plasma flows through the hollow pores [4]. The hepatocytes can remove toxins from blood or plasma, and synthesized and metabolized materials can be transported back into the body. Application of hollow fiber bioreactors has been successful in animal models of ALF and partly successful in humans with ALF [5,6]. However, an obvious disadvantage of this type of configuration is the heterogeneous distribution of hepatocytes in extra-capillary spaces. Another deficiency of this type of bioreactor is the location of the semipermeable membrane between cells and blood or plasma, which serves as a barrier to diffusion. In addition, aggregation of many hepatocytes potentially could occlude the pores of hollow fibers, which would hamper bidirectional mass transport and reduce the efficiency of the BAL devices [3,7].

To overcome the limitations of bioreactors based on hollow fiber membranes, fluidized bed bioreactors incorporating encapsulated hepatocytes have been developed as an advanced alternative [8,9]. In the recent fluidized bed bioreactors, alginate/chitosan (AC) microcapsules have been applied for hepatocyte immobilization, because this material provides a favorable three-dimensional environment for cell survival and function, and offers stability, biocompatibility, and simplicity in production [10,11]. In addition, the level of immunoisolation achieved by encapsulation of the hepatocytes in AC microcapsules may prevent injury to the encapsulated cells by the host’s immune cells [12]. In fluidized bed bioreactors, the direct frictional and impact forces of the perfusion, which cause the microcapsules to move and rise, are less than that in fixed bed bioreactors. Moreover, the spherical structure of microcapsules provides a high ratio of surface area to volume in dynamic perfusion [13], which increases the contact area to facilitate greater exchange efficiency. Also in support of greater mass exchange, the biphasic mixture achieved in fluidized bed reactors can increase the exposure of the microcapsule surfaces to the surrounding fluid [14].

Despite the noted advantages of fluidized bed bioreactors for a BAL system, further development is still needed to overcome several shortcomings of this kind of bioreactors. First, with exposure to dynamic perfusion, the contained microcapsules suffer deformation and damage from the high perfusion velocity due to their limited stiffness or strength, and such damage results in loss of immunoisolation and even hepatocyte death [7,15,16]. Damage to a high percentage of microcapsules will likely result in diminished performance of the BAL device, and methods for reducing or avoiding damage to microcapsules and hepatocytes have yet to be explored extensively. Second, a large void volume between microcapsules in the fluidized bed, which can occur even at a low flow rate, limits the space utilization and exchange efficiency [1]. Still, a sufficient flow rate is crucial for good mixing of microcapsules within the fluid to achieve mass transfer and oxygen exchange in the BAL device [17,18]. In addition to a potential increase in the void volume, with an increase in the flow rate, microcapsules may accumulate near the top of the container while deformed and broken microcapsules may be washed out and released into the medium or blood, which further compromises the safety of the BAL device. Based on these issues, a conventional fluidized bed bioreactor with a large perfusion velocity is incapable of offering the exact performance specifications required for an effective BAL device.

To circumvent the limitations of conventional fluidized bed reactors for BAL systems, we developed a diversion-type microcapsule-suspension fluidized bed bioreactor (DMFBB), in which turbine guide vanes were positioned at the bottom of a cylindrical reactor configuration to suspend microcapsules dynamically. In this study, we compared the performance of our DMFBB, in terms of fluidization, microcapsule damage, and hepatocyte function, to that of a conventional fluidized bed bioreactor (FBB) using hepatocytes of the C3A cell line entrapped in AC microcapsules.

## Materials and Methods

### Bioreactors

**Fig 1:**
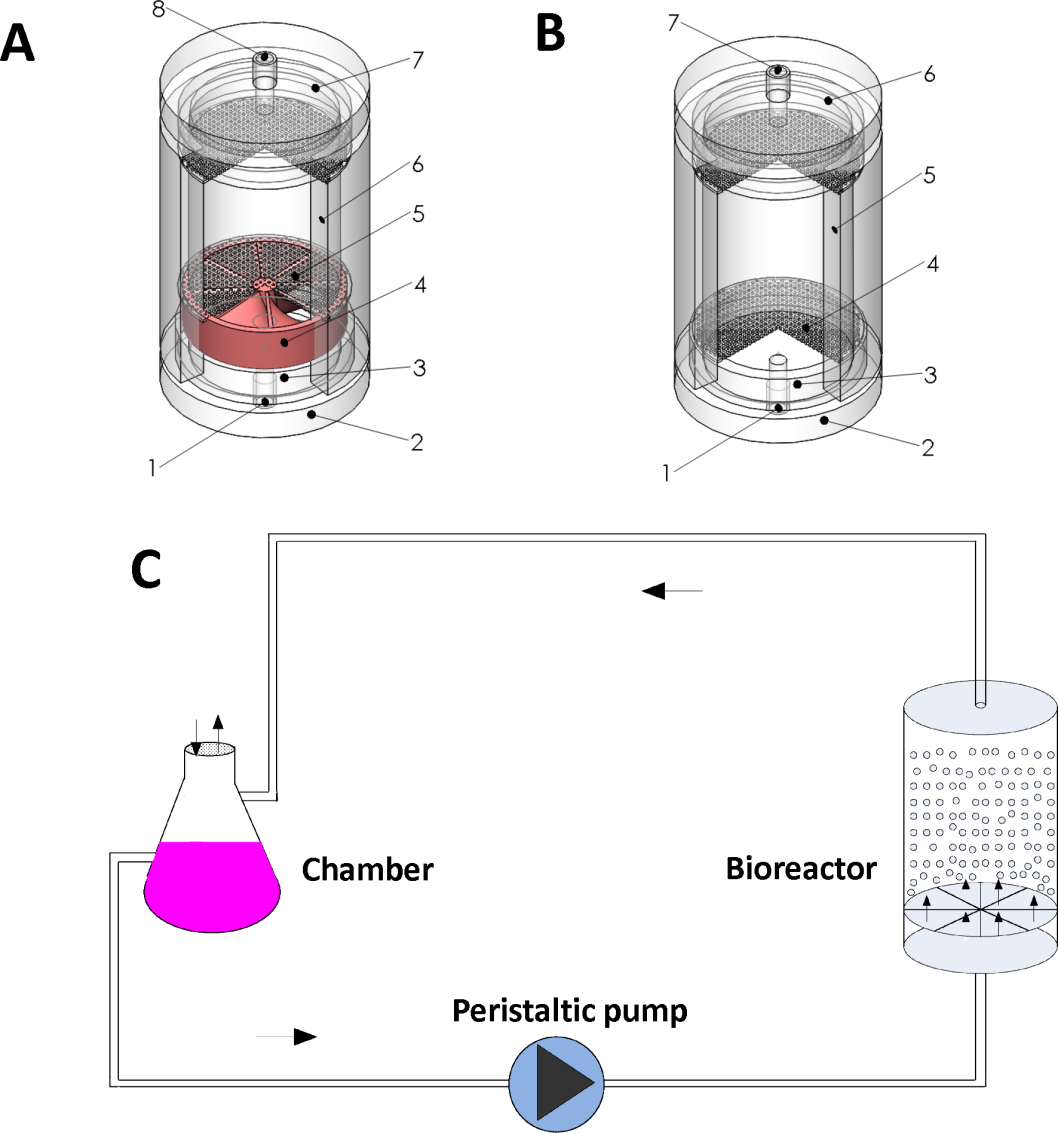
Two types of bioreactors and experimental setup for fluidization. (A) Schematic of the diversion-type microcapsule suspension fluidized bed bioreactor (DMFBB). (1) inlet; (2) bottom cap; (3) pool of incoming buffer; (4) turbine guide vanes; (5) 300 mesh membrane filters; 6) cylindrical bioreactor containing the microcapsules; (7) end cap; and (8) outlet. The dimensions associated with the turbine guide vanes were: height of the turbine = 15 mm; thickness of each turbine blade = 10 mm, blade inlet angle = 90°, blade outlet (outer edge) angle = 26°, blade outlet (inner edge) = 71°, screw pitch = 70 mm; number turbine vane rotations = 0.207, and diameter of fixed middle axis = 7 mm. (B) Schematic of the traditional fluidized bed bioreactor (FBB). (1) inlet; (2) bottom top; (3) pool of incoming buffer; (4) 300 mesh membrane filters; (5) cylindrical bioreactor containing the microcapsules; (6) end cap; and (7) outlet. (C) The experimental set up for dynamic culture of C3A cells within the fluidized bed bioreactor. C3A cells were encapsulated in alginate/chitosan microspheres with a diameter of 800 μm.

The DMFBB is a cylindrical column containing AC microcapsules, and a schematic is provided in Fig 1A. The bioreactor was characterized by eight symmetrical turbine guide vanes fixed at the bottom of the column and the following dimensions: cylinder diameter = 50 mm, cylinder height = 90 mm, and height of turbine vanes = 15 mm. The total volume was approximately 180 ml. Membrane filters of 300 mesh were attached to the bottom and top to prevent microcapsules from leaving the column. The cylinder and turbine vanes were constructed of biocompatible materials.

The conventional FBB used for comparison in this study was produced with dimensions consistent with those of the DMFBB, except that no turbine guide vanes were fixed at the bottom of the cylinder (Fig 1B).

### Microcapsule production

AC microcapsules were prepared as described previously [19]. Briefly, 80 ml 2.0% sodium alginate solution (154 mM NaCl, 10 mM HEPES, pH 7.4) was sprayed at a flow rate of 9.5 mL/min through an electrostatic microencapsulator unit (NiscoEngineering, Zurich, Switzerland). The alginate droplets fell into 0.7% chitosan solution (Jinan Haidebei Marine Bioengineering Co. Ltd, Jinan, China, powder: DAC 85.6%, viscosity 30, dissolved in deionized water containing 0.1 M CaCl_2_, and 15.6 M HEPES, pH 6.0), and were then allowed to gel for 30 min before being washed with normal saline three times. This procedure resulted in the preparation of 80 ml of empty microcapsules with a diameter of 800 μm.

### Fluidization experiment

To evaluate the performances of the bioreactors under fluidized conditions, an in vitro simulation experiment in the absence of hepatocytes and medium or plasma was performed. A buffered saline solution (137 mM NaCl, 10 mM HEPES) at 20°C was replaced of plasma, because the viscosity of the solution at 20°C was similar to that of plasma at 37°C [16]. In the experimental set-up for fluidization, the DMFBB and FBB, each containing 40 ml empty AC microcapsules, were separately connected in closed-loop circuits with peristaltic pumps (Fig 1C). As the flow rate was increased from 0 to 150 ml/min through the closed system, the AC microcapsules in the bioreactors gradually began to move, and the heights of the fluidized beds within the bioreactors increased. A millimeter ruler was directly attached to the outside of each bioreactor cylinder and used to measure the height of the fluidized bed within the bioreactor.

Fluidized bed expansion is an important parameter representing the performance of fluidization [9] and is expressed in terms of porosity (ε), which is calculated using the following equation [15]:

$$Ɛ=1-(1-{Ɛ}_{0})\frac{h_{0}}{h}$$ (1)

which can be rearranged as

$$\frac{1-{Ɛ}_{0}}{1-Ɛ}=\frac{h}{h_{0}}$$ (2)

In this equation, ε_0_ and h_0_ indicate the initial porosity and height, and h indicates the height achieved with different flow velocities. Accordingly, h/h_0_ describes the bed expansion upon fluidization. Because the initial height was fixed (20 mm), the measured height can be determined.

### Microcapsule stability

The mechanical stability of microcapsules under two different flow rates (90 ml/min and 150 ml/min) was evaluated and compared between the two types of bioreactors. The DMFBB and FBB were each loaded with 40 ml empty microcapsules and connected into the close pump circulation system for operation at the specified flow rate for 3 continuous days, stopping only for daily sampling. The collected samples were returned to the bioreactors after analysis each day.

The stability of microcapsules was evaluated by the retention rate and swelling rate after fluidization in the fluidized beds. The mechanical strength of the microcapsules was also used to evaluate microcapsule stability. Specifically, the deformation at rupture was measured after samples were maintained on a horizontal shaker.

Before fluidization and at the sampling time point each day, the total volume of empty microcapsules was measured for the two bioreactors and two flow rate conditions separately. Afterward the volume measurement, the number of microcapsules within one-fortieth of the total volume was visibly counted in order to assess the total number of microcapsules remaining in each bioreactor. The retention rate of microcapsules was determined for each condition, and each measurement was repeated three times.

Next, one-tenth of the total volume of microcapsules was removed and divided into 10 parts. Every part was placed under a light microscope, and 10 digital photomicrographs were captured at random locations using a digital camera attached to the microscope. The diameters of all microcapsules in these 100 photographs were measured. Each measurement was repeated three times. The measured diameters were used to calculate the swelling rate (S_w_), as an indicator of microcapsule stability, using the following equation (according to the relationship between volume and diameter):

$$s_{w}(100\%)\;=100\left[{\left(\frac{D_{t}}{D_{0}}\right)}^{3}-1\right]$$

In this equation, D_0_ indicates the average original diameter, and D_t_ indicates the average diameter of microcapsules after fluidization.

Finally, 500 microcapsules were counted from each group and placed under a light microscope in order to count the number of cracked and deformed microcapsules per day. Next, all 500 microcapsules were placed into a 15ml centrifuge tube with 5 ml normal saline and shaken at 180 rpm for 24h on a horizontal shaker. After 24 h, the 500 microcapsules were removed for counting of broken microcapsules again. Then, the number and percentage of broken microcapsules in each group before and after shaking were determined. Each measurement was repeated three times.

### Cell culture and encapsulation

C3A cells (CRL-10741, ATCC, Manassas, VA, USA) were cultured in Dulbecco’s Modified Eagle Medium (12430, Gibco, Auckland, NZ) supplemented with 10% fetal bovine serum (FBS) (10099, Gibco, Grand Island, NY, USA) and 1% penicillin/streptomycin (Gibco, Auckland, NZ). For collection, cells were removed from culture plates by enzymic digestion with 0.05% trypsin–EDTA(25300; Gibco, Auckland, NZ), counted, and finally diluted to 3×10^6^ cells/ml in 2.0% alginate solution. AC microcapsules were prepared as described above, except that for cell encapsulation, 120 ml of 2.0% alginate solution containing 3×10^6^ C3A cells/ml alginate was dropped into 0.7% chitosan solution to prepare 120 ml of C3A cell-laden AC microcapsules. Finally, the total volume (120 ml) of microencapsulated C3A cells was divided into three equal-volume, 40-ml aliquots, each containing 1.2×10^7^ C3A cells.

### Evaluation of bioreactor performance

To compare the performance of the DMFBB to that of the FBB, three conditions were established: (1) the DMFBB was loaded with 40 ml AC microcapsules containing C3A cells (n = 5 tests); (2) the FBB was loaded with 40 ml AC microcapsules containing C3A cells (n = 5 tests); and (3) 40 ml AC microcapsules containing C3A cells was maintained in static medium as the control (n = 5 tests). For all three conditions, the experimental systems were kept in an incubator with an internal temperature of 37°C and a gas mixture of 95% air and 5% CO_2_ for the 72h experimental period. The DMFBB and FBB were connected into the closed circulation system with a final volume of 200 ml culture medium and operated at a flow rate of 150 mL/min. Samples were taken from each system for analysis every 24 h.

### Cell viability

Cell viability was evaluated using the MTT assay (Roche, Basel, Switzerland; 11465007001) according to the manufacturer’s instructions. Briefly, 20 microcapsules were placed into a well of a 96-well plate containing 100 μl culture medium, and 10 μl MTT labeling reagent was added per well and incubated for 4 h. Then 100 ml solubilization solution was added to each well and incubated overnight. The formation of purple formazan crystals was measured using a microplate reader (DTX880; Beckman Coulter, Brea, CA, USA) at wavelength of 570 nm.

Cell viability was also assessed by cell staining. Live cells in microcapsules were labeled with fluorophoresrhodamine 123 (R123), 5- (and 6-) carboxy-4',5'-dimethylfluorescein diacetate (CMFDA,C7025, Eugene, OR, USA), and dead cells were labeled by propidium iodide (PI, 556463, Sigma, USA) [20]. Briefly, 1 ml microcapsules were placed into a well of 24-well plate containing 300 μl culture medium, 3 μl CMFDA, and 3μl PI and incubated for 10 min. Then the live and dead cells were identified, and photomicrographs were captured under a fluorescence microscope (IX81, OLYMPUS, JAPAN).

### Cytochrome P450 1A2 and 3A4 activity assays

Cytochrome P450 1A2 and 3A4 enzyme activity was evaluated in 24 well plates directly by testing luciferase activity with the P450-Glo CYP1A2 assay (V8422; Promega, Madison, WI, USA) and CYP3A4 assay (V9002; Promega). Briefly, cells in 1 ml microcapsules were incubated at 37°C in Krebs–Henseleit buffer containing Luciferin-1A2 or fresh medium containing Luciferin-IPA for 1 h. Then 50 μl buffer or culture medium was removed from each well and transferred to a 96-well opaque white plate and mixed with 50 μl luciferin detection reagent. After incubation for 20 min at room temperature, luminescence was measured using a microplate reader (DTX880; Beckman Coulter).

### Urea and albumin synthesis tests

The urea concentration was determined using a Urea Assay Kit (DIUR-500, Biotechnology BioAssay Systems, Hayward, CA, USA), and the albumin concentration was determined using the Human Albumin ELISA Quantitation Kit (E80-129; Bethyl Laboratories, Montgomery, TX, USA). All results were analyzed using CurveExpert 1.3 software and fitted with a logistic regression model with r^2^>0.99.

### Real-time polymerase chain reaction (PCR) analysis

At the end of the 72 h experimental period, C3A cell-containing microcapsules were dissolved in 55 mM Na_3_C_6_H_5_O_7_-2H_2_O. After centrifugation at 1,000 rpm for 5 min, cells were washed with phosphate-buffered saline twice [21]. RNAs were extracted using TRIzol reagent (15596026, Invitrogen, Carlsbad, CA, USA) according to the manufacturer’s instructions. cDNAs were synthesized using oligo primers and a reverse transcription kit (205311; Qiagen, Hilden, Germany). Real-time quantitative PCR analyses were performed on a Bio-Rad Cycler using customized PCR arrays with various sequences. The primers used in this study are listed in Table 1.

**Table 1:** Primers Used in the Real-time Quantitative PCR Analyses.

Name	Forward	Reverse	Gene bank
GAPDH	GCACCGTCAAGGCTGAGAAC	TGGTGAAGACGCCAGTGGA	NM 002046.3
CYP1A2	CTGGGCACTTCGACCCTTAC	TCTCATCGCTACTCTCAGGGA	NM 000761.3
CYP2B6	TCTGGCCGGGGAAAAATCG	GGTCACAGAGAATCGCCGAAG	NM 000767.4
CYP2C8	GGAAAACGAATTTGTGCAGGAG	GTGGCAGAGAAACAATCCCTT	NM 001198855.1
CYP2D6	CCAACGGTCTCTTGGACAAAG	GGGTCGTCGTACTCGAAGC	NM000106.4
CYP3A4	AGATGCCTTTAGGTCCAATGGG	GCTGGAGATAGCAATGTTCGT	NM 057096.2
CYP3A5	GCAAACAGCCCAGCAAACA	GTCCATCGCCACTTTCCTTC	NM 001190484.1
CYP2E1	GATGCCCTACATGGATGCTG	AAATGGTGTCTCGGGTTGCT	NM 000773.3
UGT1A1	TCCCACTTACTGCACAACAAG	GGTCCGTCAGCATGACATCA	NM 000463.2
UGT2B4	CAAATGTTGAGTTCGTTGGAGGA	CTGACGTGTTACTGACCATCG	NM 021139.2
HNMT	GTGGAAAAAGTACGGATCACGC	GGCATTAAAGTTGCAGGTTTCAG	NM 006895.2
ALB	GATGCCTGCTGACTTGCCTTC	TCAGCAGCAGCACGACAGAGTA	NM 000477.5
GST	ACCATCCCTTTGGCTATTGAGA	TTCTGCCTGCGGAGTTTATCA	NM024751.2

### Statistical analysis

Measurements are presented as mean ± standard deviation (SD) values. Statistical analysis was performed using Student’s t-test and one-way analysis of variance (ANOVA) with SPSS for Windows Version 16.0 (SPSS, Inc., Chicago, IL, USA). Differences were considered statistically significant if *p* <0.05.

## Results

### Comparison of fluidization in the DMFBB and FBB

**Fig 2:**
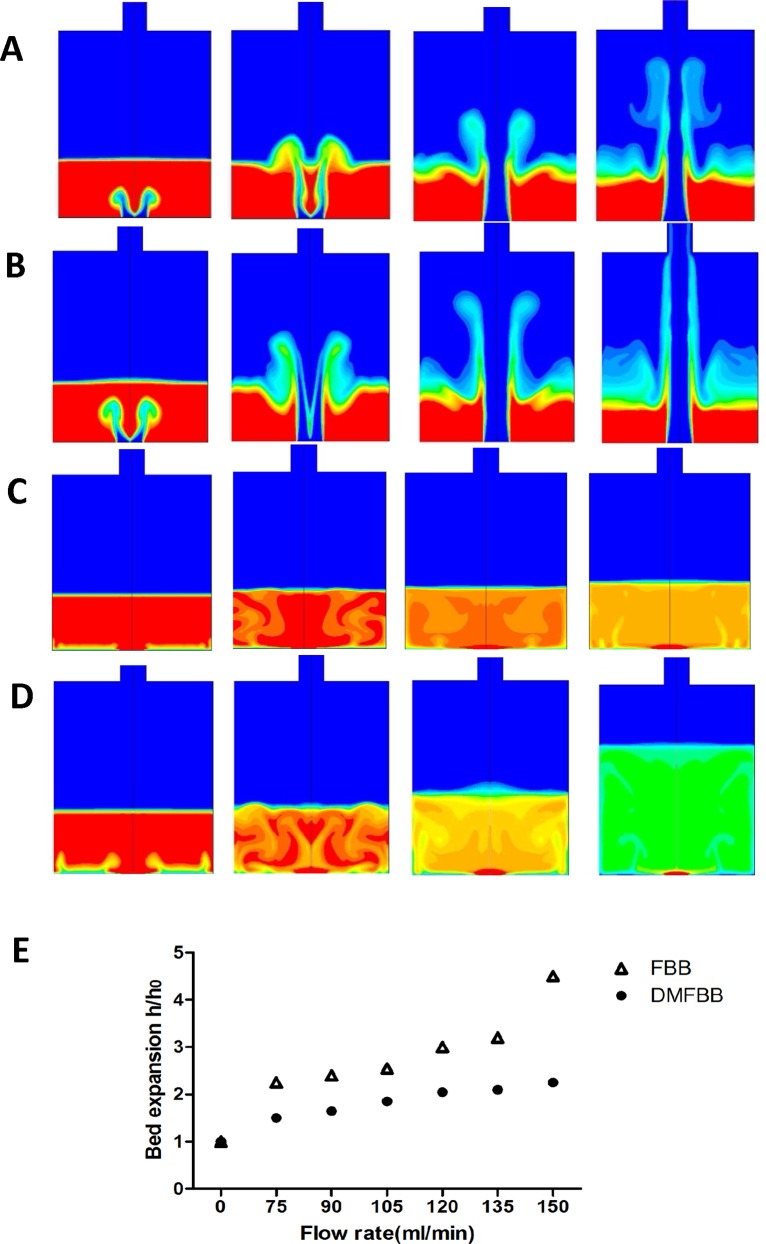
Fluidization performance of two bioreactors. (A) Fluidization performance of the FBB at 90 ml/min. (B) Fluidization performance of the FBB at 150 ml/min. (C) Fluidization performance of the DMFBB at 90 ml/min. (D) Fluidization performance of the DMFBB at 150 ml/min. At the beginning, the red indicates fixed microcapsules at the bottom of the reactor, and the blue indicates fluidized flow (DMEM). As the flow rate was increased, microcapsules in the center at the bottom of the FBB were forced upwards to the top of the bioreactor, leading to weak fluidization. Conversely, the microcapsules in the DMFBB were gradually mixed with the flowing medium, and eventually, dynamic and balanced fluidization was established. In the images, the colors from red to yellow or green represented different microcapsule densities under different conditions. (E) Fluidization in DMFBB and FBB in terms of bed expansion (h/h0) as the perfusion flow rate was increased from 0 to150 ml/min.

Different fluidization phases were built up in the DMFBB and FBB from the fixed to final stable fluidized beds. In the FBB, a portion of the microcapsules in the center of the column were pushed by the perfusion flow and forced upward along the central axis sharply, whereas the microcapsules away from the center or at the edge of the container were exposed to weak perfusion. As a result, fluidization in the control bioreactor exhibited a disequilibrium state and a disproportionate distribution of microcapsules. However, in the DMFBB, because the flow force of the inlet perfusion was shunted into a diversion-type vortex by the turbine guide vanes at the bottom of the bioreactor, the fluidized bed within the DMFBB was found to rise horizontally and uniformly as a whole as the microcapsules were lifted by the increasing flow velocities. Gradually, dynamic fluidization was established. The different fluidization performances of the FBB and DMFBB at different flow rates of 90 and 150 ml/min were evaluated at several time points as shown in Fig 2.

In addition to the visual inspection of the performances of the two bioreactors, the bed expansion (h/h0) results at different flow rates demonstrated the behavior of fluidization in both bioreactor types (Fig 2E). At every flow rate, the height of the bed in the FBB was higher than that in the DMFBB. The sharp increase in the bed expansion within the FBB from 2.4 to 4.5 as the flow rate increased from 90 to 150 ml/min, compared to the corresponding increase from 1.65 to 2.25 in the DMFBB, indicates that greater bed expansion occurred in the FBB than in the DMFBB during fluidization.

### Comparison of microcapsule damage in the DMFBB and FBB

**Fig 3:**
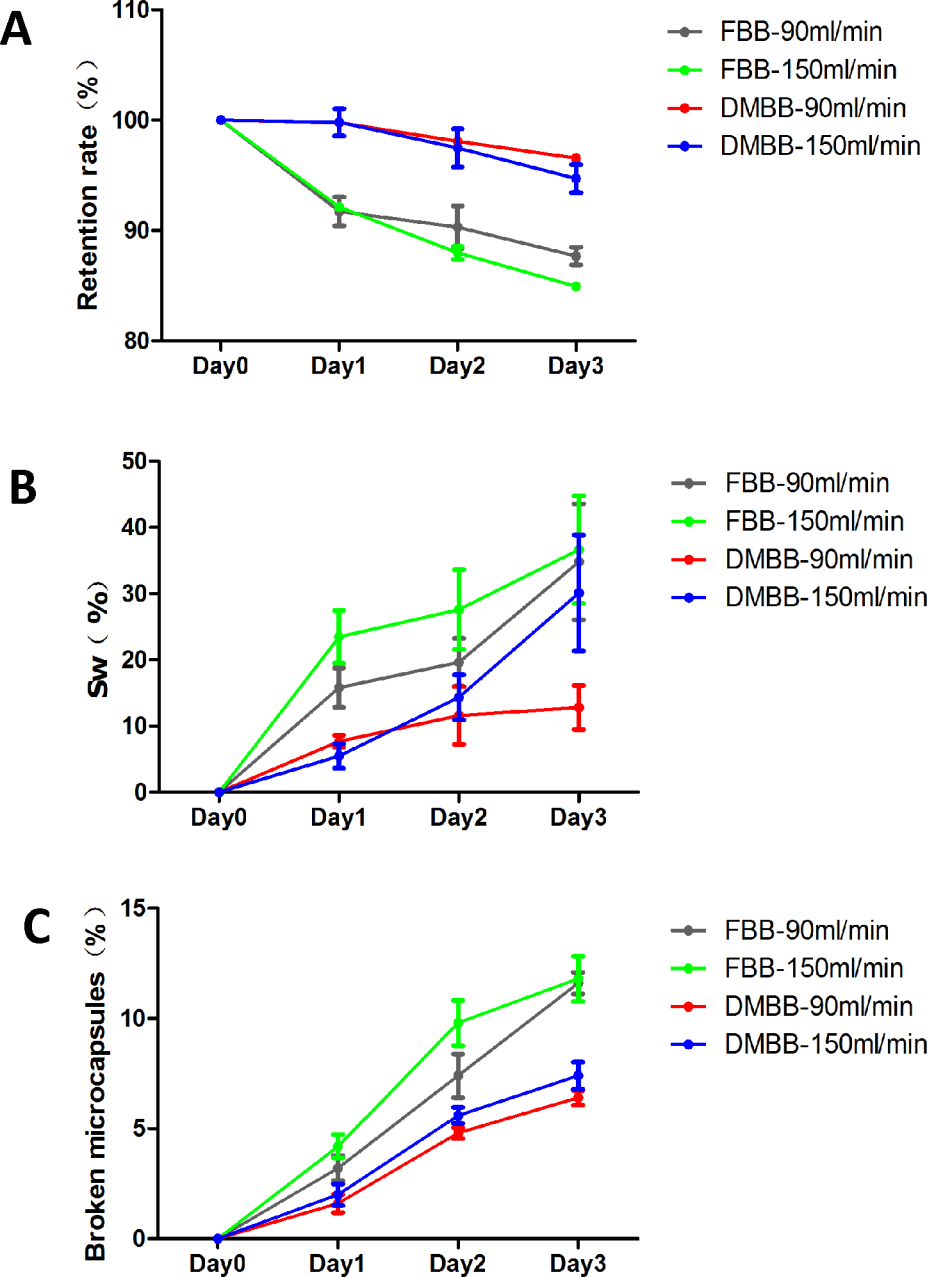
Effects of fluidization on empty microcapsule integrity within the bioreactors. (A) Microcapsule retention rates of the DMFBB and FBB operated at 90 and 150 ml/min. (B) Swelling rates (%) of microcapsules in the DMFBB and FBB operated at 90 and 150ml/min. (C) Percentages of broken microcapsules in the DMFBB and FBB operated at 90 and 150 ml/min. The following results were obtained: (A): When the DMFBB was operated at 90 ml/min, the rate of microcapsule retention was 99.8% compared to 91.74% in the FBB at day 1 (p = 0.0025), 98.08% compared to 90.3% at day 2 (p = 0.0022), and 96.55% compared to 87.68% at day 3 (p = 0.0024).When the DMFBB was operated at 150 ml/min, the rate of microcapsule retention was 99.78% compared to 92.14% in the FBB at day 1 (p = 0.0051), 97.49% compared to 87.98% at day 2 (p = 0.0014), and 94.71% compared to 84.95% at day 3 (p = 0.0008). (B): When the DMFBB was operated at 90 ml/min, the swelling rate (%) of microcapsules was 7.7% compared to 15.81% in the FBB at day 1 (P = 0.0176), 11.59% compared to 19.64% at day 2 (p = 0.0027), and 12.8% compared to 34.81% at day 3 (p = 0.0005). When the DMFBB was operated at 150 ml/min, the swelling rate of microcapsules was 5.49% compared to 23.49% in the FBB at day 1 (p = 0.0328), 14.35% compared to 27.59% at day 2 (p = 0.0241), and 30.11% compared to 36.66% at day 3 (p = 0.3258). (C): When the DMFBB was operated at 90 ml/min, the percentage of broken microcapsules in the DMFBB was 1.6% compared to 3.2% in the FBB at day 1 (p = 0.0095), 4.8% compared to 7.4% at day 2 (p = 0.0117), and 6.4% compared to 11.6% at day 3 (p = 0.0017). When DMFBB was operated at 150 ml/min, the percentage of broken microcapsules in the DMFBB was 1.9% compared to 4.2% in the FBB at day 1 (p = 0.0042), 5.6% compared to 9.8% at day 2 (p = 0.0114), and 7.4% compared to 11.8% at day 3 (p = 0.0031).

Microcapsule damage was assessed in both bioreactor types at flow rates of 90 and 150ml/min according to the microcapsule retention rate (Fig 3A), swelling rate (Fig 3B), and rate of microcapsule breakage after shaking (Fig 3C).

Between each experimental time point, the decrease in the retention rate of microcapsules in the DMFBB was not obvious from the previous 2 days at each flow rate (*p*>0.05), unlike the significant decrease in the retention rate in the FBB at each flow rate and time point (*p*<0.05). The differences in the decrease in the retention rate between the two flow rates (90 to 150 ml/min) were not significant for each bioreactor type (*p*>0.05). However, the retention rate of microcapsules in the DMFBB was significantly greater than that in the FBB at every time point for the same flow rate (*p*<0.05).

The rate of microcapsule swelling followed a similar trend between the bioreactor types and flow rates (Fig 3B). The increase in swelling rate over time in the DMFBB was not significant in comparison to that on the previous 2 days (*p*>0.05), and the rate of microcapsule swelling in the DMFBB was always less than that in the FBB in each condition (*p*<0.05). Notably, the rate of microcapsule swelling was significantly greater in the DMFBB when the flow rate was 150 ml/min compared to 90 ml/min (*p*<0.05).

Before and after application of shaking force for 24 h, except the broken rate in the DMFBB at day 1 for two flow rates, the percentages of broken microcapsules increased over time in each condition (*p*<0.05, Fig 3C). However, between the two flow rates, the percentages of broken microcapsules did not differ significantly for each bioreactor type (*p*>0.05). Similar to the other indicators of microcapsule damage, the percentage of broken microcapsules in the DMFBB was significantly lower than that in the FBB at every time point (*p*<0.05).

Overall, these results indicate that significantly less microcapsule damage occurred in the DMFBB compared to the FBB over 72 h of fluidization at different flow rates.

### Comparison of cell viability in fluidized and static conditions

**Fig 4:**
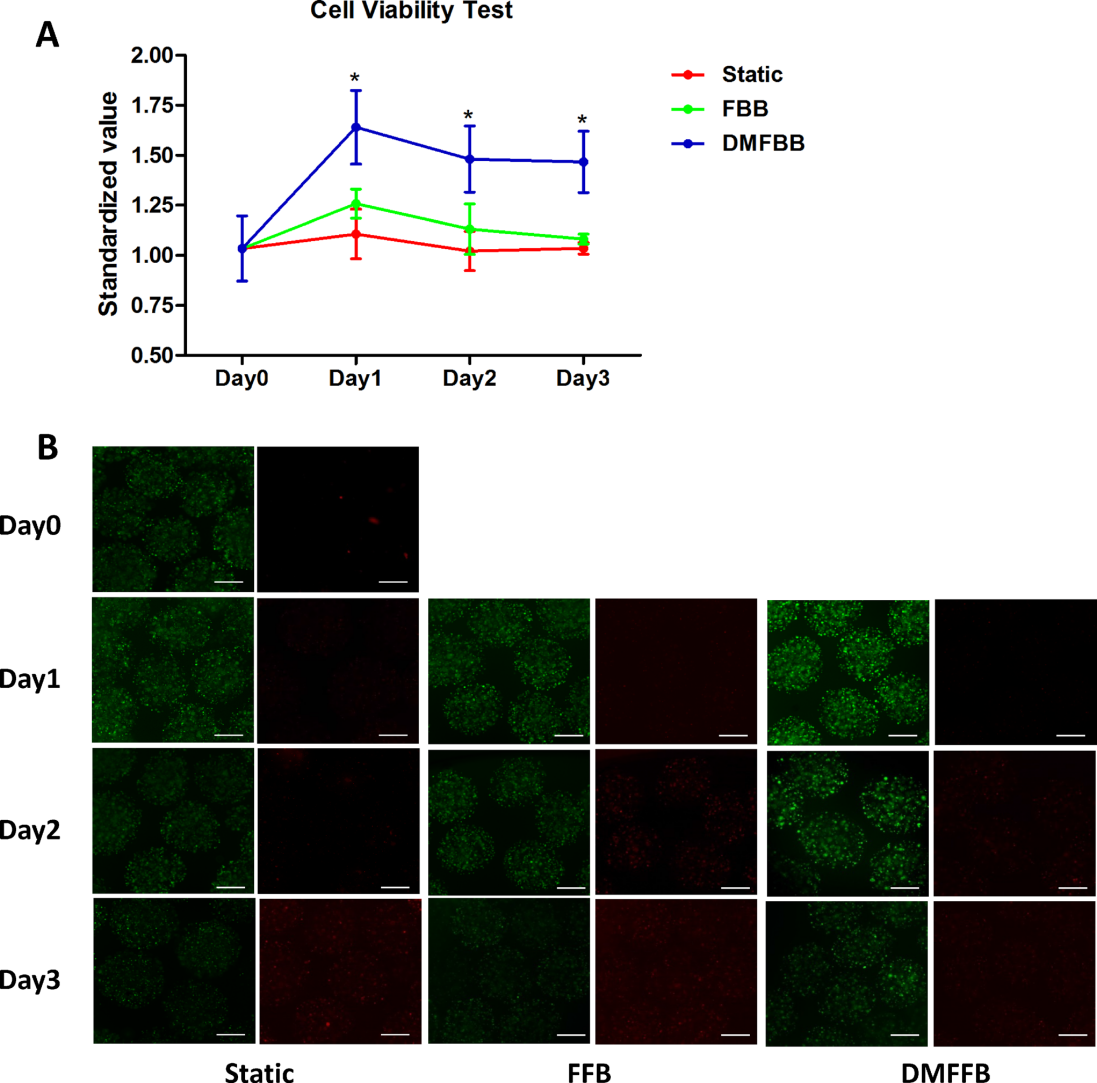
C3A cell viability in fluidized and static culture conditions. (A) Cell viability according to MTT assay results; *p<0.05. Cell viability in the DMFBB was significantly improved compared to that in the FBB on each day of the 3-day experiment (p = 0.0155, 0.0098, and 0.0112). The differences between the FBB and static group were not significant over the 3-day time course (p = 0.0987, 0.0512, and 0.3086). (B) Images of live (CM-FDA stained) and dead (propidium iodide stained) C3A cells in microspheres. Scale bar, 500 μm.

According to the results of MTT assays (Fig 4A), the metabolic activity of C3A cells increased significantly in the DMFBB during the first day of operation and then remained stable over the subsequent 2 days. In contrast, no significant changes in cell activity were observed in the FBB or in the static control conditions during the 3-day experimental period. As a result, cell viability in DMFBB was higher than that in the FBB and static culture (*p*<0.05) at each time point, whereas the differences between the FBB and static conditions were not significant (*p*>0.05).

Fluorescent staining for live and dead cells within AC microcapsules collected from each condition provided results consistent with those of the MTT assays (Fig 4B).Qualitative visual observation of live cells under fluorescence microscopy showed more live cells in microcapsules from the DMFBB than in those from the FBB and the static culture condition.

### Comparison of hepatocyte function in fluidized and static conditions

**Fig 5:**
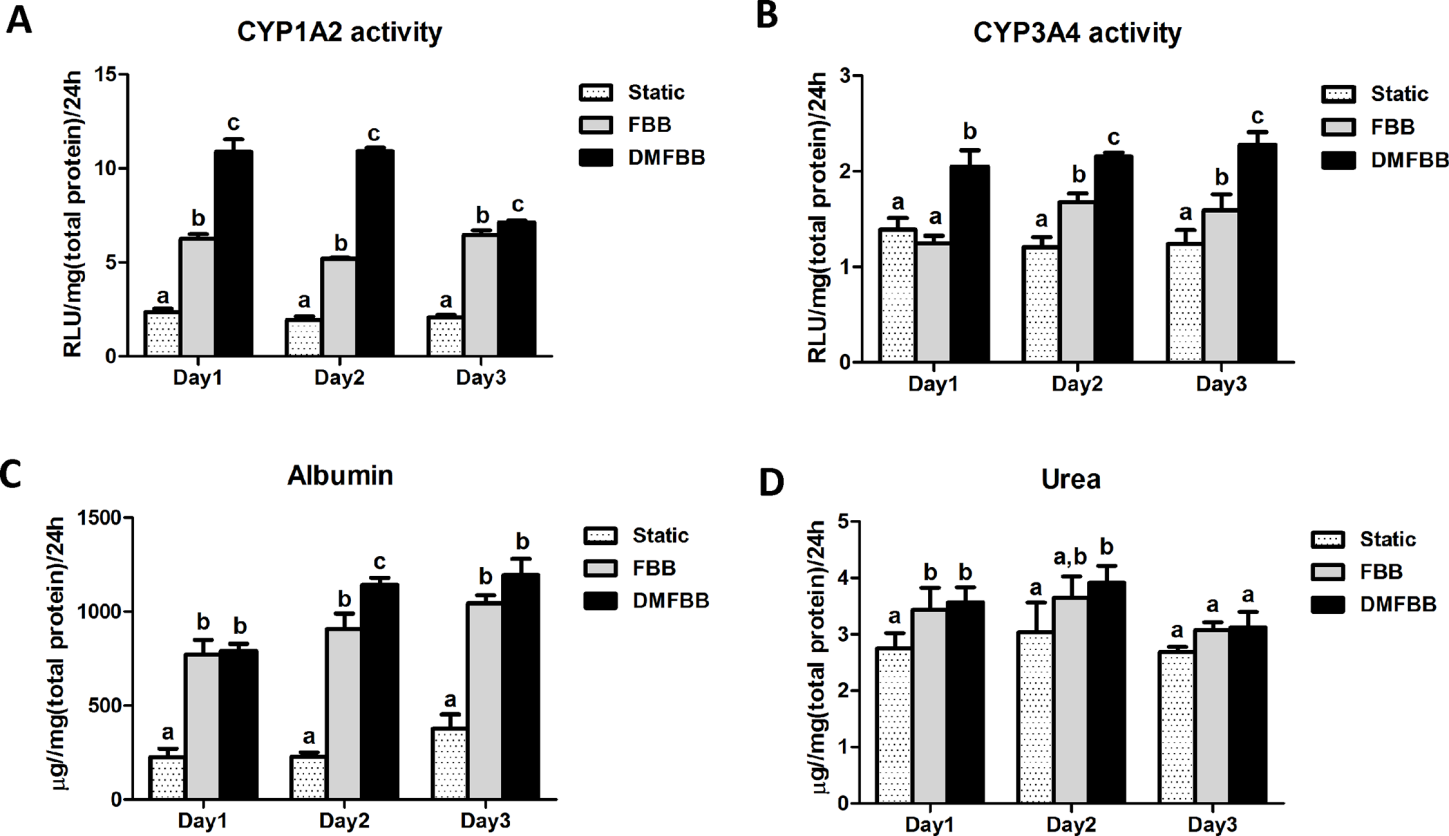
Hepatocyte-related functions of C3A cells in fluidized and static culture conditions. (A and B) Activities of phase I enzymes CYP 1A2 (A) and CYP3A4 (B) measured via fluorometric substrates in different fluidized and static culture conditions. (C and D) Rates of albumin secretion (C) and urea synthesis (D) of C3A cells in different fluidized and static culture conditions. Columns labeled with the same letter indicate the results were not statistically different; for all other comparisons, *p*<0.05.

The activities of the main phase I enzymes (P450 CYP1A2 and CYP3A4), which are essential to the effectiveness of the BAL, were tested to evaluate the biotransformation ability of C3A cells in fluidized and static culture conditions (Fig 5A and 5B). As expected, the activities of the main CYP450s were significantly increased when C3A cells were cultured in both types of fluidized bed bioreactors. In additional, the CYP1A2 and CYP3A4 activities of C3A cells in the DMFBB were higher than those of C3A cells in the FBB, indicating that encapsulated C3A cells in our newly developed fluidized bed bioreactor achieved a higher detoxification capacity.

The albumin concentration (Fig 5C) as a measure of its secretion by C3A cells and the urea concentration (Fig 5D) as a measure of its synthesis by C3A cells were quantitatively measured to determine the maintenance of biosynthesis functions of the encapsulated cells. The albumin concentrations in the two fluidized bioreactors were significantly greater than that in the static culture condition (*p*<0.05), and the difference in the albumin concentration between the DMFBB and FBB was statistically significant on day 2 *(p*<0.05). The function of urea synthesis by the encapsulated C3A cells was also greater with culture in fluidized conditions for the first 2 days (*p*<0.05), but this improvement in the fluidized conditions was much less obvious on the third day of the experiment.

### Comparison of gene expression in hepatocytes in fluidized and static conditions

**Fig 6:**
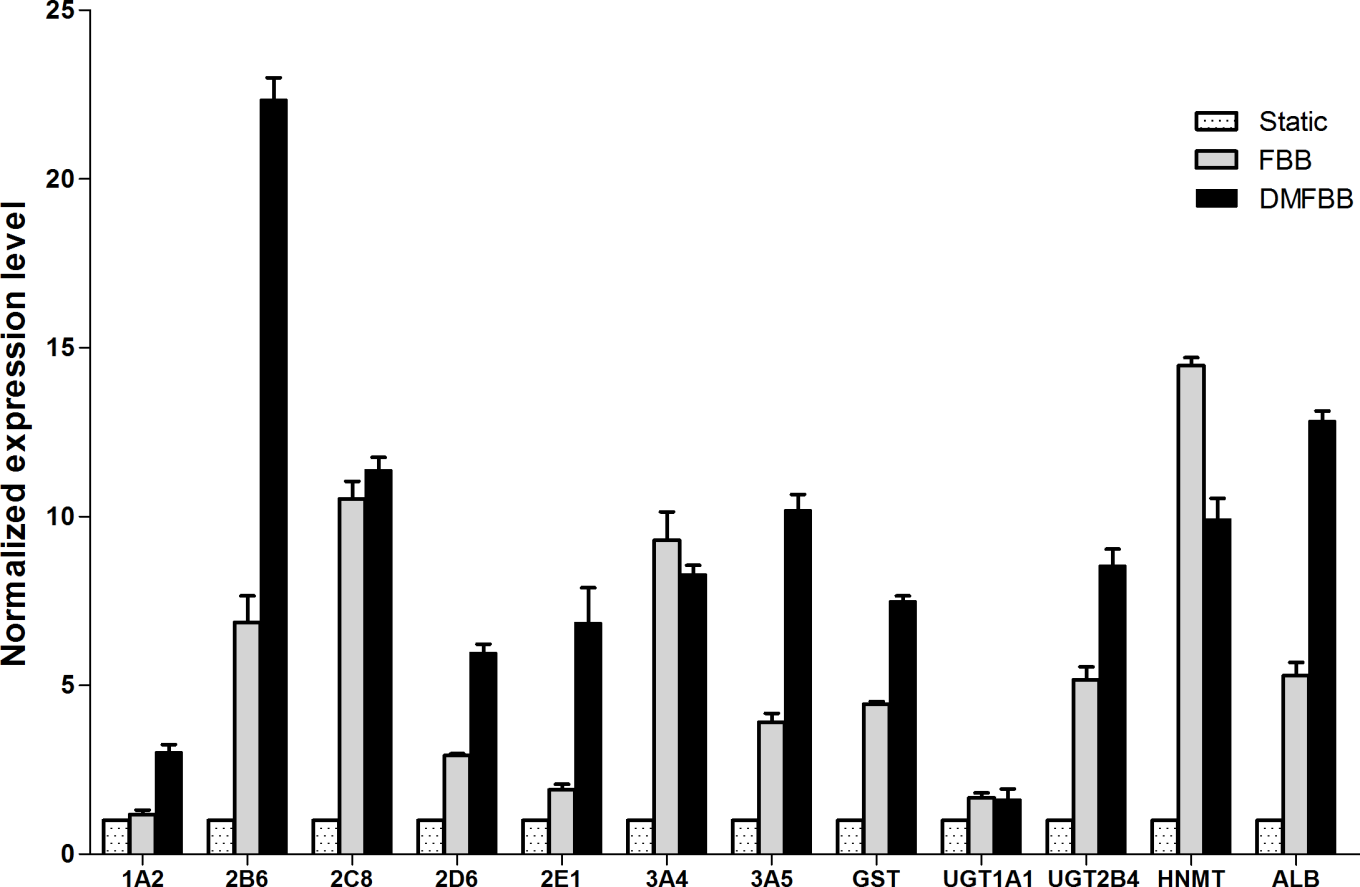
Normalized gene expression of CYP450 and phase II enzymes in C3A cells after 72 h of culture in fluidized and static conditions. All data were normalized to the activity of C3A cells cultured in static conditions.

Several classes of CYP450 phase I and phase II enzymes and specific proteins [22] were selected to test whether the DMFBB could improve the transcription of metabolism-related genes in hepatocytes by real-time PCR (Fig 6). The normalized transcription levels of several CYP450-related genes and albumin-related genes (1A2, 2E1, 2B6, ALB, GST, 3A5, 2D6, and UGT2B4) were significantly greater in C3A cells in the DMFBB compared to levels in C3A cells in the FBB and in static culture. In addition, the expression levels of 3A4 and 2C8 in both bioreactor types were higher than those in static culture, but the differences in these levels between the DMFBB and FBB were not significant. In contrast, the normalized expression of UGT1A1 and HNMT by C3A cells in the DMFBB was less than that by cells in the FBB (*p*<0.05). Overall, these results indicated that many classes of genes were differentially expressed in C3A cells cultured in the DMFBB.

## Discussion

Although multiple studies have shown that fluidized bed bioreactors can be adopted for BAL systems used to effectively treat patients with liver failure [23,24], research aimed at improving the performance of these bioreactors for specific use in BAL systems is lacking. In the present study, we develop a new fluidized bed bioreactor incorporating AC encapsulated hepatocytes with the goals of decreasing the void volume and reducing microcapsule damage. We also studied the performance of our fluidized bed bioreactor in terms of encapsulated hepatocyte viability and function.

The microcapsule diameter might influence the properties or biological characteristics of microcapsules with or without cells [25]. In our previous study, microcapsules of different diameters (300 μm and 800 μm) were evaluated in terms of empty microcapsule permeability and viability of hepatocytes contained within microcapsules. The results showed that there were no significant differences between microcapsules of the two diameters (S1 Fig). Because BAL systems must support large-scale cell culture, microcapsules with a large diameter will be beneficial to efficient production, owing to greater simplicity in preparation. Furthermore, previous research showed that microcapsules with a large diameter can offer favorable permeability and maintain cell viability, indicating that cytokines and growth factors could be sufficiently exchanged within these microcapsules [19,21]. In a study by Gautier et al. [26], considering nutrient mass transfer as well as cell viability and function, beads with a large diameter of 1000 μm offered a reliable entrapment process for hepatocytes to be used in a fluidized bed bioartificial liver. As a result, microcapsules with a large diameter of 800 μm were employed in our study.

We assessed the void volume with the bioreactor according to bed expansion. Void volume has previously been expressed as the dead volume in which there is no available access to cells [27,28], and a high void volume has an obvious effect on the mass transport between cells and the perfused solution [29]. We found that bed expansion in the DMFBB was significantly less than that in the conventional FBB, indicating the void volume was less in the DMFBB. With this lower void volume, microcapsules were allowed to achieve desirable fluidization in the DMFBB, which should not only reduce the dead volume in the bioreactor but also improve the contact space and mixing efficiency between hepatocytes and the perfused solution.

In the human liver, blood is generally processed at a flow rate of 1500 mL/min, whereas a flow velocity of at least 100–300 mL/min is considered appropriate for in vitro testing of BAL devices for technical and rheological reasons [30]. However, a high flow velocity is associated with a high shear force that could result in greater damage of microcapsules within bioreactors [31,32]. Because the microcapsules in which hepatocytes are encapsulated provide a proper barrier to protect hepatocytes from the host immune response, preservation of microcapsule integrity helps to avoid the loss of hepatocytes in BAL devices [12,33]. Thus, decreasing or avoiding extensive damage to microcapsules and loss of hepatocytes can increase the effectiveness of a bioreactor designed for use in a BAL system.

Shear force has been considered one of the most decisive factors in the formation of the dynamic state within a fluidized bed bioreactor [34], with exposure to excessive shear force inducing microcapsule damage and reducing the performance of the bioreactor [35]. However, shear force within a bioreactor is often difficult to describe due to the complex patterns of fluid flow [36]. In our study, higher microcapsule retention rates as well as reduced microcapsule swelling and breakage were observed in the DMFBB compared to the conventional FBB. We propose that this reduction in microcapsule damage was achieved by exposure of the microcapsules to a more uniform perfusion flow at all levels and directions as achieved by the symmetric turbine guide vanes at the bottom of the bioreactor, which likely created a low shear force environment for the microcapsules. Importantly, mechanical stimulation conveyed by the fluid flow in terms of moderate shear force in the DMFBB, which might mimic the forces during in vivo blood flow, has been acknowledged as crucial impetus to provide better control of mass delivery [37]. Such improved maintenance of the stability of microcapsules in the DMFBB could provide favorable conditions for preserving adequate numbers of healthy and functioning hepatocytes for use in a BAL system. In the conventional FBB, the microcapsules were subjected to a greater shear force caused by nonuniform perfusion across the bioreactor and thus showed a higher degree of the deformation and breakage.

In addition to affecting shear forces within the fluidized bed, a appropriate bed expansion with fluidization is conducive to homogenous mixing under optimized hydrodynamic conditions that can be beneficial for mixing cells with medium completely and thereby increasing the availability of cells for nutrient/oxygen delivery, metabolite exchange, and waste elimination [8,14,15]. Good transport of these factors can enhance cell viability and function, and in our study, we observed such improvements in cell viability and some functions related to synthesis and detoxification metabolism in C3A cells cultured in the DMFBB in comparison to those cultured in the FBB and in static conditions. Finally, in addition to greater contact of the cells with the surrounding medium, a more uniform cell distribution can contribute to the equilibrium of fluidization in the bioreactor [38–40]. Together, the results of the current study imply that the more uniform distribution of not only shear force but also perfusion medium throughout the DMFBB may support its potential for superior performance compared to conventional FBBs. Previous studies have also shown that under improved fluidization conditions, medium perfusion through the bioreactor helped dictate the hydrodynamic shear forces that lead to altered gene expression profiles and functional changes [31,32,41]. In our study, conditions in the DMFBB promoted the metabolic and synthesis functions of hepatocytes, specifically as related to detoxification via activities of CYP450 enzymes and phase I/II metabolism. Consistently, increased activity of CYP450 enzymes has been shown to up-regulate genes related to phase I/II metabolism [42]. As expected, efficient detoxification, which is vital to the performance of BAL systems [41], resulting from the increased transcription of liver-specific genes as well as greater CYP450 activity. Although the gene expression of CYP3A4 in the DMFBB was slightly less than that in the FBB, this difference was not statistically significant. Notably, many factors influence gene transcription and subsequent translation. The phenomena of superinduction and upregulation refer to an increase in the concentration of a protein [43,44]. In this study, CYP3A4 activity in the different groups could be affected by many regulating factors, leading to the high concentrations in the DFMBB even without an obvious increase in CYP3A4 gene expression. However, the mechanisms underlying the functional improvements and regulation of gene expression levels in encapsulated hepatocytes within the different bioreactors remain to be determined in future studies.

## Conclusion

In this study, we developed a novel DMFBB and evaluated the effectiveness of its design for decreasing void volume, protecting microcapsule integrity, and promoting C3A cell viability and function in AC microcapsules under fluidized conditions. Our findings indicate that the developed DMFBB provides a promising alternative to current in vitro flow chambers and offers the added advantage of being able to culture more hepatocytes due to the lower void volume achieved and greater stability of microcapsules within the bioreactor. Thus, the DMFBB appears to be suitable for practical application in BAL systems, but scale up will be required for use in animal experiments and clinical trials for treating acute hepatic failure or other severe liver diseases.

## Supporting Information

S1 Fig Permeability and cell viability within AC microcapsules with diameters of 300 μm and 800 μm. **(A) Permeability within AC microcapsules with diameters of 300 μm and 800 μm.** Concentration of BSA in microcapsules with diameters of 300 μm and 800 μm. **(B) Cell viability within microcapsules over 3 days according to MTT assay.** Columns labeled with the same letter indicated the results were not statistically different, p>0.05 (p = 0.79, 0.18, 0.21, and 0.59).
(TIF)

S2 Fig HE staining and scanning electron microscopy (SEM) of microencapsulated hepatocytes. **(A) HE staining showing a uniform distribution of hepatocytes with dark stained nuclei and no obvious necrosis. Scale bar, 200 μm. (B) SEM images of encapsulated cells showing:** (a) cell nucleus at low magnification; (b) cell nucleus and organelles at high magnification; (c) microvilli and cell junctions; (d) mitochondria, endoplasmic reticulum, and ribosomes; and (e) the structure of microvilli.
(TIF)
