# Supplementary figures and images for: A New Fluidized Bed Bioreactor Based on Diversion-Type Microcapsule Suspension for Bioartificial Liver Systems
Source: PLoS One. 2016 Feb 3;11(2):e0147376. doi: 10.1371/journal.pone.0147376 (PMC4739599; doi:10.1371/journal.pone.0147376)

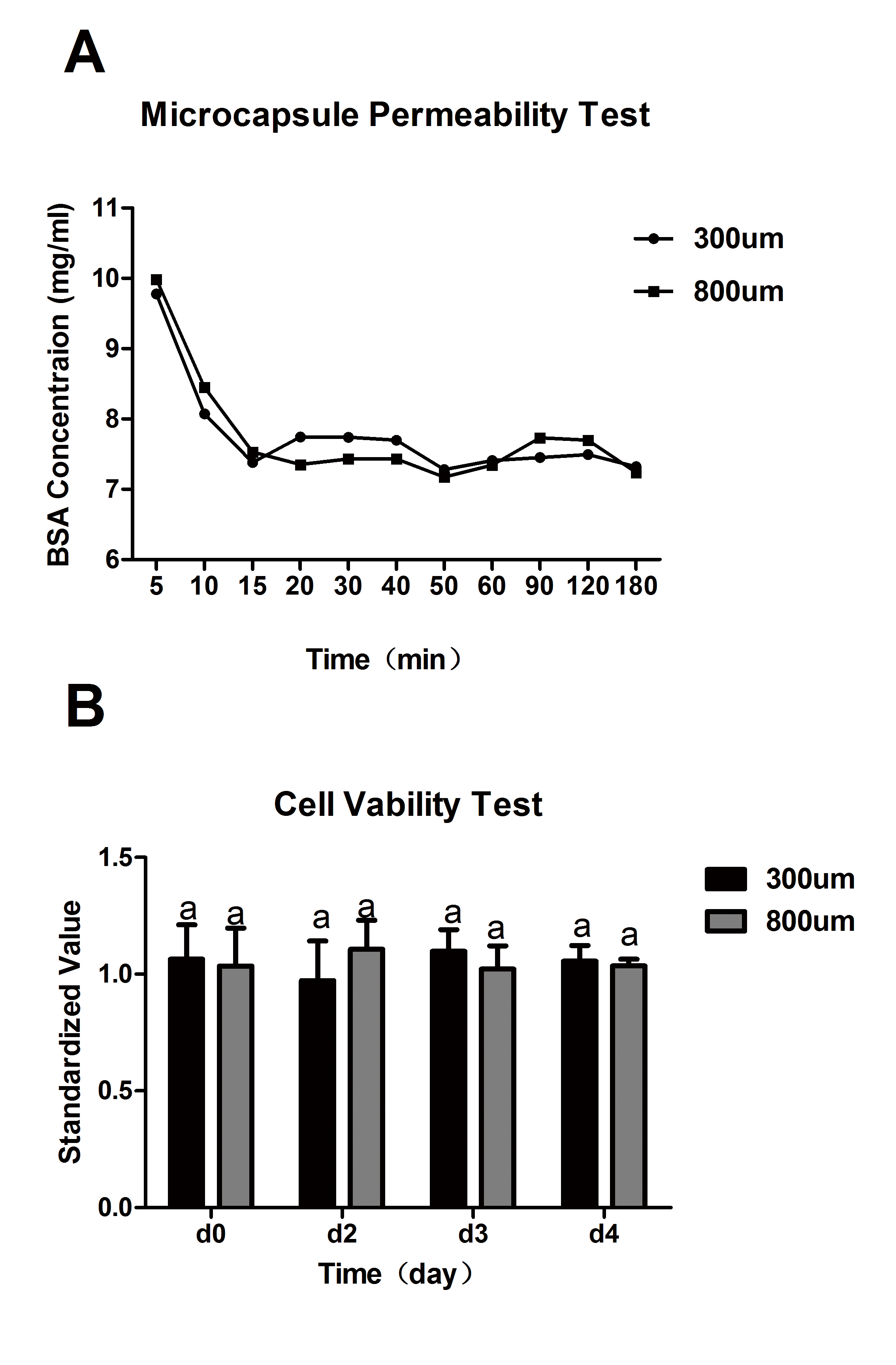

Supplement: S1 Fig — (A) Permeability within AC microcapsules with diameters of 300 μm and 800 μm. Concentration of BSA in microcapsules with diameters of 300 μm and 800 μm. (B) Cell viability within microcapsules over 3 days according to MTT assay. Columns labeled with the same letter indicated the results were not statistically different, p>0.05 (p = 0.79, 0.18, 0.21, and 0.59). (TIF) [file pone.0147376.s001.tif]

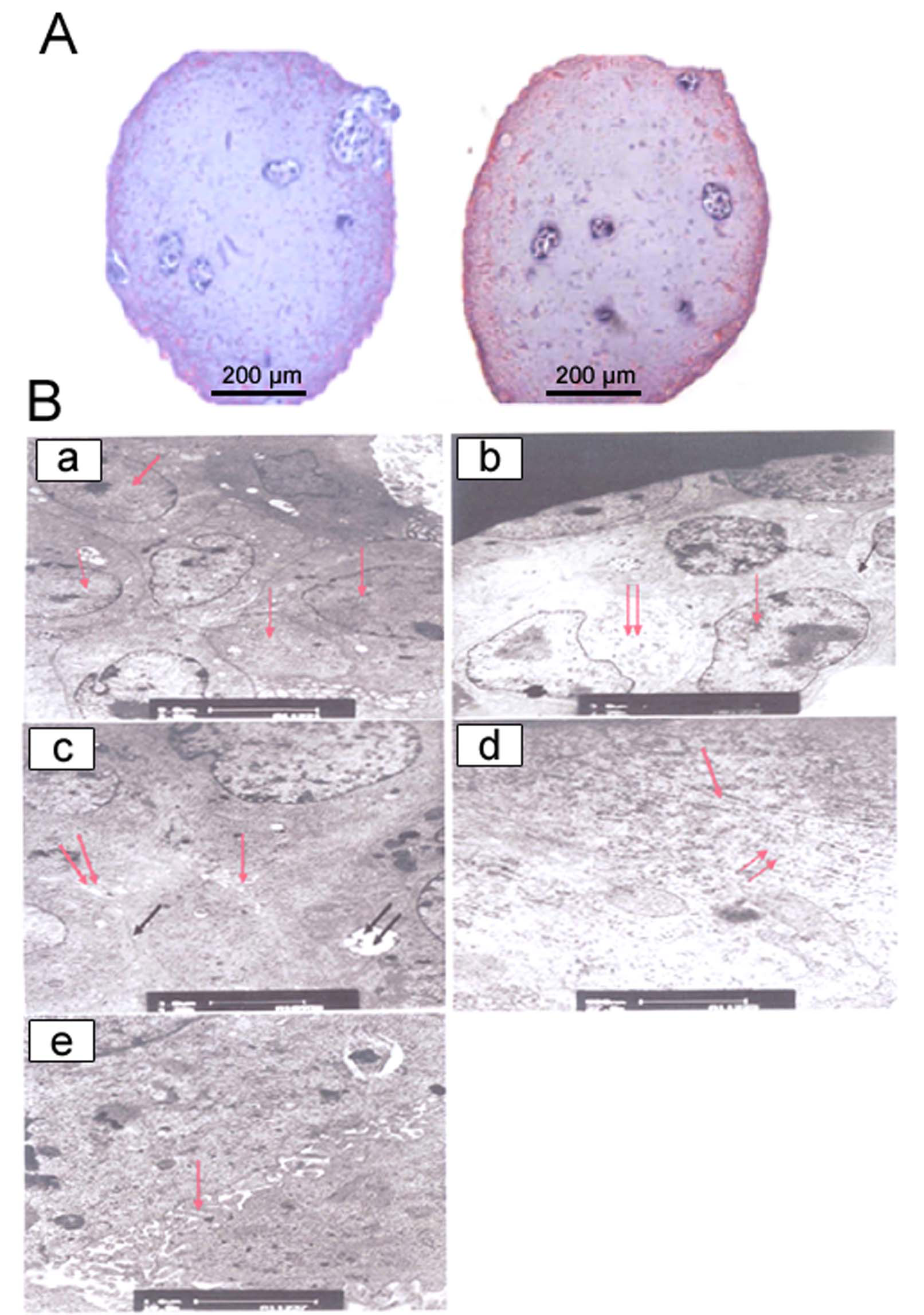

Supplement: S2 Fig — (A) HE staining showing a uniform distribution of hepatocytes with dark stained nuclei and no obvious necrosis. Scale bar, 200 μm. (B) SEM images of encapsulated cells showing: (a) cell nucleus at low magnification; (b) cell nucleus and organelles at high magnification; (c) microvilli and cell junctions; (d) mitochondria, endoplasmic reticulum, and ribosomes; and (e) the structure of microvilli. (TIF) [file pone.0147376.s002.tif]
